# Paving the Way to Precision Nutrition Through Metabolomics

**DOI:** 10.3389/fnut.2019.00041

**Published:** 2019-04-09

**Authors:** Abdellah Tebani, Soumeya Bekri

**Affiliations:** ^1^Department of Metabolic Biochemistry, Rouen University Hospital, Rouen, France; ^2^Normandie Univ, UNIROUEN, CHU Rouen, INSERM U1245, Rouen, France

**Keywords:** metabolomics, precision nutrition, biomarker, omics, precision medicine

## Abstract

Nutrition is an interdisciplinary science that studies the interactions of nutrients with the body in relation to maintenance of health and well-being. Nutrition is highly complex due to the underlying various internal and external factors that could model it. Thus, hacking this complexity requires more holistic and network-based strategies that could unveil these dynamic system interactions at both time and space scales. The ongoing omics era with its high-throughput molecular data generation is paving the way to embrace this complexity and is deeply reshaping the whole field of nutrition. Understanding the future paths of nutrition science is of importance from both translational and clinical perspectives. Basic nutrients which might include metabolites are important in nutrition science. Moreover, metabolites are key biological communication channels and represent an appealing functional readout at the interface of different major influential factors that define health and disease. Metabolomics is the technology that enables holistic and systematic analyses of metabolites in a biological system. Hence, given its intrinsic functionality, its tight connection to metabolism and its high clinical actionability potential, metabolomics is a very appealing technology for nutrition science. The ultimate goal is to deliver a tailored and clinically relevant nutritional recommendations and interventions to achieve precision nutrition. This work intends to present an update on the applications of metabolomics to personalize nutrition in translational and clinical settings. It also discusses the current conceptual shifts that are remodeling clinical nutrition practices in this Precision Medicine era. Finally, perspectives of clinical nutrition in the ever-growing, data-driven healthcare landscape are presented.

## Introduction: Metabolomics and Metabotypes

The metabolome defines the metabolites present in a given organism (1). Metabolomics refers to the analysis of the metabolome of biological system (2, 3). Metabolites are small organic molecules involved or not in enzymatic reactions. Thus, metabolomics is a technology that aims to biochemically characterize a metabolome and its changes regarding genetic, environmental, drug, or dietary factors (4). Hence, metabolomics is an appealing tool to define metabotypes (metabolic phenotypes) that could be used for individual stratification. It is well known that metabolic characteristics drive individual differences regarding nutritional requirements and responses to diet and medication (5, 6). Thus, based on this metabolic specificity, dietary recommendations could be tailored at the individual level. This high customization is supposed to be more effective in terms of health-care outcomes and costs than conventional general recommendations (5, 7). These endeavors led to the emergence of the concept of metabotyping or metabolic phenotyping, which stratify individuals based on their metabolic and phenotypic patterns into metabotype subgroups with high metabolic similarity. Thus, this metabolic similarity could be used for population stratification to define tailored dietary or medical intervention (8–10). Two main technologies are used in metabolomics, Nuclear magnetic spectroscopy (NMR) and mass spectrometry (MS) combined or not with a gas phase or liquid phase separation method (11). NMR spectroscopy has the advantage to be non-destructive, rapid, highly reproducible, and robust but it lacks sensitivity. MS-based methods are more sensitive, but they are destructive and might have lower throughput especially if combined with a separation dimension. These techniques recover global, unbiased, and comprehensive chemical information from the assessed biological samples. Metabolomics workflows are based on the previous knowledge of the metabolites to analyze. A targeted approach is defined as a quantitative analysis when absolute concentrations are determined or a semiquantitative analysis when relative intensities are determined for a set of metabolites. The untargeted approach is primarily based on the qualitative or semiquantitative analysis of the most possibly detectable metabolites in the biological sample. Untargeted metabolomics is often used for biomarker discovery studies. Depending on the study, different parameters should be taken into account; defining the metabolomics approach (targeted vs. untargeted), biological samples, sample size, pooling, experimental conditions (i.e., observational studies, exploratory studies, time series), sampling conditions and storage, analytical platforms, and standardized sample preparation procedures. To translate the metabolomics data into actionable information, data analysis. The choice of the most appropriate data analysis strategy depends mostly on the underlying investigation aim. In mechanistic studies, the structure of the data descriptions of the built model are more important than its predictive power to classify new samples. In diagnosis or prognostic applications, the classification performances of the model are crucial. It is also to be noted that sample sizes and model validation using external validation sets are mandatory to avoid overfitting of the models to avoid spurious conclusions (12). In this review we will focus on the use of metabolomics in human studies to achieve personalized nutrition or precision nutrition (PN) which refers to the design of customized nutritional recommendations to prevent or to treat nutrition-related disorders (13).

## Pathways Toward Precision Nutrition

### Nutritional Research

Nutritional research focuses on unveiling the interrelationships between diet and health to prevent or treat diet-related disorders (14). It is established that the individual nutritional needs vary largely according to anthropometrics such as age, gender and also on physiological states and other biological and environmental attributes (15). Furthermore, the response to dietary interventions exhibits substantial inter-individual variation. In this era of precision medicine, it is time to tailor dietary intervention and advice at the individual level such as biomarkers related to red meat (16) or bread consumption (17). To effectively understand diet and lifestyle interaction in health and disease, robust, and informative biomarkers for nutritional status are mandatory (18, 19). However, different challenges hamper the application of nutritional biomarkers. First, there is no consensus on nutritional biomarker definition, its assessment and use. Furthermore, even the well-validated biomarkers lack consistency to support strong recommendations (20, 21). To overcome these constraints, several initiatives have been launched to promote the identification and development of new nutritional biomarkers by defining methods, conditions, and settings of nutritional biomarker quantification to set standardized and actionable protocols (22, 23). In addition to this organizational and strategic endeavors, deep and extensive research is still mandatory to provide a deep molecular understanding of nutrients effects on health and disease. Recently, a comprehensive definition of personalized nutrition has emerged that describes three main levels in precision nutrition: ***level 1*** is dietary advice based on dietary intake; ***level 2*** adds to level 1 phenotypic metrics and laboratory tests; ***level 3*** goes deep by adding, to the previous levels, the genetic information layer (22). Large-scale initiatives such as the Food4me performed randomized control trial to assess improvements in personalized dietary advice compared to conventional approaches (7). The results showed changes in dietary behavior related to customized nutritional advice. However, from a public health perspective, these results are hard to scale up. This work intends to present an update on the applications of metabolomics to personalize nutrition in translational and clinical settings. It also discusses the current conceptual shifts that are remodeling clinical nutrition practices in this Precision Medicine era.

### Traditional Dietary Assessment Techniques and Their Limitations

For decades, nutritional research has been a crucial pillar to unveil diet–health relationships at both individual and population scale. However, consistency, validation and reproducibility of the dietary assessments have been the great challenges (19). For a long time, food diaries, 24 h recalls and Food Frequency Questionnaire (FFQ) are used as self-report dietary assessment methods in human nutrition studies (24). Multiple days weighted food records are often used in controlled dietary studies (25). However, these dietary records are time consuming and tedious for both participants and investigators, and lack accuracy and consistency (25, 26) which limits their integration in large-scale studies. Alternatives such as 24 h recalls may be used. This method may include a structured collection of detailed information about food intake during the previous 24 h. A major drawback is that these recalls are subjected to inter-day nutritional variation (25). For large-scale studies, self-administered FFQ are preferred to evaluate food intake. All these methods (food records, recalls, and FFQ) require an intensive preparation before implementation. However, the managing and processing of a well-validated FFQ is rather smooth for large studies. Still, inaccurate portion size estimation, socially distorted answers, lack of objectivity and accuracy in self-reported data, and errors in food intake composition hamper FFQ and make it prone to record errors (27, 28). The implementation of high-throughput omics tools enables the holistic and integrative interrogation of nutrition by identifying nutrition-specific biomarkers to objectively assess dietary intake. These biomarkers may provide actionable information to fill in the gaps of self-reported food intake given the tight connection between metabolite production, food intake, the microbiome, and health status (29). Indeed, stratification of individuals into groups can be achieved through the analysis of their respective metabolic profiles. This approach of grouping subjects based on their metabolic phenotype was coined “metabotyping” and has been used in several of studies (30, 31). This review will examine this concept in the field of personalized nutrition.

### Pathways Toward Precision Nutrition

The goal of precision or personalized nutrition (PN) is the design of customized nutritional recommendations to prevent or to treat nutrition-related disorders (13). PN aims to set dynamic nutritional recommendations based on individual attributes and external environmental factors. Hence, PN strategies should include genomics information, other factors such as dietary and physical activity patterns, metabolome, and microbiota. The post-genomic era generated various genome-wide association studies (GWAS) that allow the identification of genomic factors that might unveil the inter-individual metabolic response variability to specific diets. Various genes and polymorphisms have been defined as relevant factors to explain diet-specific metabolic responses (32–36). However, there is weak clinical evidence to establish comprehensive and integrated framework for personalized nutritional interventions in clinical settings (37). Despite this, different successful implementations already made it to the clinic. For example, in inborn errors of metabolism (IEM) which are a group of about 500 rare genetic diseases due to defects in a given biochemical pathway due to the deficiency or abnormality of an enzyme, its cofactor, or a transporter, leading to an accumulation of the substrate or lack of the product. In IEM, nutritional strategies are crucial in treatment of some disease such as phenylketonuria screening (38). Interventional nutrition has allowed the implementation of specific diet and saved patients from devastating outcomes for them and their families. In the wellness sphere, genetic tests are used to define slow or fast metabolizers (33). While genomic-based customized nutrition is already being implemented, PN might lack sufficient evidence for full integration into clinical setting (39).

## Metabolomics as a Key Enabler of Precision Nutrition

Metabolomics is a key tool to investigate the effect of food on the individual's health. By identifying food-derived biomarkers, inter-individual variability in metabolizing same foods in health and disease states. Metabolomics could enhance nutrition assessment through three main pathways: dietary intake biomarker discovery, diet-related diseases exploration through cohort studies, and dietary intervention assessment through metabolic patterns. A summary of discussed studies are presented in Table 1.

**Table 1:** Examples of metabolomics-based studies in nutritional research.

**Groups**	**Platform**	**Aim**	**Main findings**	**References**
**DIETARY INTAKE BIOMARKER DISCOVERY USING INTERVENTION STUDIES**				
8 healthy males 18–50 years old	Fluorescence detection Targeted metabolomics	To compare the effects of citrus dietary levels of proline betaine on glycine betaine excretion, and on plasma total homocysteine and betaine concentrations in healthy volunteers	Proline and betaine in dietary levels had little effect on plasma total homocysteine concentrations in healthy humans.	(40)
17 healthy males 24–74 years old	Fluorescence detection Targeted metabolomics	Four analytes (creatinine, taurine, 1-methylhistidine, and 3-methylhistidine) specifically found in meat and excreted in urine were investigated.	Urinary 1-methylhistidine and 3-methylhistidine as potential biomarkers of meat intake	(41)
7 females and 1 male 28–45 years old	NMR untargeted metabolomics	Identification of coffee consumption biomarkers	2-furoylglycine as a putative biomarker for coffee consumption	(42)
20 healthy males 18–40 years old	NMR untargeted metabolomics	Nutrikinetic modeling of tea consumption using metabolic data	Identification of increased urinary excretion of several gut-mediated metabolites of tea flavonoids	(43)
280 males and 285 females 18–64 years old	NMR untargeted metabolomics	To identify urinary biomarkers indicative of sugar-sweetened consumption	Formiate, citrulline, taurine, and isocitrate were identified as markers of sugar-sweetened beverages intake	(44)
35 healthy males 18–69 years old	Untargeted and targeted metabolomics Mass spectrometry and NMR	Identification of wine consumption biomarkers	Red wine and grape juice consumption alters microbial fermentation and amino acid metabolism	(45)
**DIETARY BIOMARKER DISCOVERY USING COHORT STUDIES**				
33 males and 35 females 58–60 years old	Untargeted mass spectrometry metabolomics	To develop a data-driven procedure to discover urine biomarkers indicative of habitual exposure to different foods	Specific metabolites as dietary biomarkers of oily fish [methyl-histidine] and coffee [dihydrocaffeic acid derivatives]	(46)
1,257 females and 790 males 35–64 years oldIncluding 801 type 2 diabetes cases	Mass spectrometry targeted metabolomics	This study aimed to identify blood metabolites that possibly relate red meat consumption to the occurrence of type 2 diabetes	Six biomarkers (ferritin, glycine, diacyl phosphatidylcholines 36:4 and 38:4, lysophosphatidylcholine 17:0, and hydroxy-sphingomyelin 14:1) were associated with red meat consumption and diabetes risk	(16)
275 males (55–80 years) and females (60–80 years)	Untargeted mass spectrometry metabolomics	Whole-grain bread consumption biomarkers	Pyrraline, riboflavin, 3-indolecarboxylic acid glucuronide, 2,8-dihydroxyquinoline glucuronide, and *N*-α-acetylcitrulline were also tentatively identified	(17)
275 males (55–80 years) and females (60–80 years)	Untargeted mass spectrometry metabolomics	To characterize the dietary walnut fingerprinting	18 metabolites, including markers of fatty acid metabolism, ellagitannin-derived microbial compounds, and intermediate metabolites of the tryptophan/serotonin pathway	(47)
**DIETARY BIOMARKER DISCOVERY USING DIETARY PATTERNS**				
24 healthy premenopausal females 20–50 years old	Targeted metabolomics	To identify typical and atypical metabolite temporal patterns in response to paired meal challenge tests.	Three subgroups related to insulin resistance and leptin levels are identified	(48)
12 males and 12 females 36–69 years old	Ultraviolet detection targeted metabolomics	Assessed the response to dietary carotenoids in juice (watermelon and tomato)	Five metabolic subgroups with related to dietary. This study identified strong and weak metabolizers of carotenoids over time	(49)
740 males and 760 females 18–90 years old	Mass spectrometry targeted metabolomics	Assessment of demographics, dietary habits, and metabotype	Two subgroups were identified regarding fasting metabolic profile and the postprandial insulin levels.	(50)
19 postmenopausal finish females 56–66 years old	Untargeted mass spectrometry	Assessment of metabolic phenotypes in a randomized controlled, crossover meal study	Medium- to long-chain acylcarnitines shows opposite patterns related to fruits and desert intake. Whereas, short-chain acylcarnitines and amino acids, were positively correlated with saturated fat	(51)
79 females 28 males 18–65 years old	Untargeted mass spectrometry	Developing a compliance tool by using metabotyping strategies by comparing average Danish Diet or a New Nordic Diet for 6 months	22 unique food exposure markers were identified that covered 7 food groups (strawberry, cabbages, beetroot, walnut, citrus, green beans, and chocolate)	(52)
16 females 21 males 18–50 years old	Targeted mass spectrometry	Assessment Western and Prudent dietary patterns on metabotypes	Western dietary pattern is related to saturated fat intakes with a metabolic signature characterized by higher levels of short-chain acylcarnitines and amino acids including branched amino acids and aromatic amino acids	(53)

### Dietary Intake Biomarker Discovery Using Intervention Studies

Dietary intervention studies are based on consuming specific food followed by biofluid collection either postprandial or following a short-term intervention (54). Urine is the most used biological sample due to its convenience compared to plasma and serum. This strategy unveiled potential food intake biomarkers such as citrus fruit (40), red meat (41), coffee (42), tea (43), sugar-sweetened beverages (44), and wine (45). Even though, several food specific biomarkers have been reported they still lack independent large-scale validation studies. It also worth noting that most of these biomarkers are rapidly secreted in urine. Thus, they are mostly acute food intake biomarkers. Hence, given this temporal limit, longer-term biomarkers of food intake are crucially needed. Currently, an increasing number of studies are assessing biomarkers in serum and plasma.

### Dietary Biomarker Discovery Using Cohort Studies

Large-scale cohort studies are also valuable tools to discover dietary biomarkers. Dietary data may be collected using traditional methods to identify consumer profiles related to specific food (e.g., low and high consumers, consumers, and non-consumers). In this case, metabolomic profiles are compared between these subgroups to unveil potential dietary biomarkers. Given their practicability, Self-reported dietary assessment methods are often used in large-scale studies tend to rely on which are known to be notoriously prone to error. It is worth noting these studies are mainly association studies and don't allow causal inference (55). Intervention studies are, therefore, needed to validate the potential metabolite as a specific food intake biomarker. Several cohort studies identified intake biomarkers such as fish (46), red meat (16, 56), whole-grain bread (17), and walnuts (47). Furthermore, metabolomics has also been used in populational stratification (57–59). Recently, Wittenbecher et al. (16) applied serum metabolomics to unveil significant relationships between different red meat intake biomarkers with risk of type-2 diabetes. The authors state that high levels of ferritin, low glycine, and altered hepatic derived lipids in the circulation were associated with both total red meat consumption and diabetes risk. This first study assessing a large set of metabolites revealed the link between red meat intake and diabetes risk and thus achieved an important step in biomarker discovery in precision nutrition. Unveiling these links between will allow deeper understanding potential metabolic pathways as disease drivers. These pathways could be validated through more interventional nutrition studies using targeted approaches for biomarker validation.

### Dietary Biomarker Discovery Using Dietary Patterns

O'Sullivan et al. pioneered the combination of dietary patterns and metabolomic patterns (60). Then, this approach has been widely used in several studies (53, 61–64). It generally involves applying chemometrics and multivariate statistical strategies to model the metabolic patterns and the food intake behavior. Different methods could be used. Unsupervised learning methods are exploratory and are used to define metabolically similar groups. The methods included k-means cluster analysis (65–70), hierarchical clustering (71–73), self-organizing maps (74), principal component analysis (PCA) (48, 51), factor analysis (70), and mixed effect modeling (75, 76). Supervised learning methods are predictive and are used to select biomarkers to predict diet related metabolic patterns. Among these methods partial least squares regression is widely used (51, 77). Univariate statistics such as *t*-test and ANOVA, can also be used to find discriminant features between the studied groups. It is worth noting that cross-validation and the use of training and validation sets are crucial for model validation. Krishnan et al. (48) used metabolomics to stratify response groups to meals with different glycemic indices. The study included 24 healthy premenopausal women aged between 20 and 50 years old. The authors assessed blood glucose, insulin, and leptin as covariates. The study revealed three distinct biologically meaningful subgroups one with higher insulin resistance and the other with higher leptin levels (48). Wang et al. assessed the response to dietary carotenoids in juice (watermelon and tomato) including 23 healthy subjects (49). Dynamic response of individual plasma carotenoids has been assessed. The study revealed five metabolic subgroups with related to dietary carotenoids. This study identified strong and weak metabolizers of carotenoids over time. The different responses seem to be induced by genetic variants of the carotenoid metabolizing enzyme β-carotene 15,15'-monooxygenase 1 (49). Li et al. (50) from the Irish National Adult Nutrition Survey (NANS) included 1,500 participants (740 males and 760 females) aged between 18 and 90 years. This study assessed habitual food and beverage consumption in an Irish cohort. The authors assessed 26 plasma fatty acids and identified four subgroups with distinct fatty acid patterns related to demographics, dietary habits, and metabotype (50). Moazzami et al. assessed metabolic phenotypes in a randomized, controlled, crossover meal study including 19 postmenopausal finish women with mean age of 61 years. One hundred eighty-nine metabolites were assessed (51). Two metabolic subgroups were identified regarding fasting metabolic profiles and the postprandial insulin levels. Despite similar glycaemia after bread intake, plasma leucine, isoleucine, sphingomyelins, and phosphatidylcholines were associated with insulin sensitivity. The authors suggest the use of fasting metabotypes to probe the postprandial insulin behaviors in individuals with normal glycaemia to stratify population (51). Andersen et al. aimed to develop a compliance tool by using metabotyping strategies (52). The authors have applied untargeted metabolomics on 181 urine samples from subject assigned to either an Average Danish Diet or a New Nordic Diet for 6 months. The data resulted in a predictive model used to track non-compliant subjects. This study shows how metabolomics could help to build metabolic predictors to follow complex diets compliance as long as metabolites are quantitatively assessed and the multivariate models are robustly validated. Dietary patterns may also help in studying diet and disease interrelationships. Bouchard-Mercier et al. studied in 37 plasma, using targeted metabolomics, the metabotypes associated with the Western and Prudent dietary patterns. The data suggest that medium- to long-chain acylcarnitines show opposite patterns related to fruits and desert intake. Whereas, short-chain acylcarnitines and amino acids, were positively correlated with saturated fat (53). These studies show the potential of metabolomics is not only a tool dietary pattern compliance, but also to identify and assess diet and disease relationships (78–80).

## Interrogating Microbiota-Diet Cross Talks Through Metabolomics

Gut microbiota profiling is gaining great interest in nutritional interventions to assess dietary effects on gut microbiome ecosystem diversity (81). The core idea is to tailor nutritional interventions by optimizing the richness and diversity of the gut microbiota (82). Diet may effect on the gut microbiota and its impact on the microbial metabolome is very appealing. Diet may serve as a substrate that can be processed by the gut microbiota for the production of small molecules to sustain host-microbiome interactions (83–85). For example, it has been reported that the majority of microbiota-derived short-chain fatty acids is absorbed by the host (86) and contributes to host nutrition (87). The FRUVEDomics Study is a behavioral interventional trial. It aims to identify metabolomics- and microbiome-based nutritional risk factors. The study included 36 participants that were randomized into three intervention groups, fruits and vegetables diet, associated either with low-fat or low carbohydrates (88). The results suggested that metabolic syndrome is correlated to higher Firmicutes to Bacteroidetes ratio (88). Other studies highlight the relevance of interrogating the gut microbiota to achieve precision nutrition (89, 90). These studies suggest that increased fasting plasma levels of trimethylamine (TMA), produced by gut microbiota, is associated with increased risk of atherosclerosis. These studies led to targeted recommendations of nutrition advice such as reducing red meat intake (91) for subjects with gut microbial ecosystem that could convert red meat related nutrients into proatherogenic molecules. Other general recommendations may also be questioned, such as using artificial sweeteners, as reported by Suez et al. (92) who reported that high sweetener intake may lead to glucose intolerance in subjects with sensitive gut microbiota (92). However, their results seem to be controversial given the used high dose of sweetener (93, 94). Another way to take advantage of this microbiota-host interaction through diet and thus, through metabolites, is to use prebiotics which are substrates that are selectively and used by host microorganisms and led to better health for the host. They are another appealing mean to take advantage of microbiome modulation (95). Some studies suggest a potential therapeutic use of prebiotics (96–99). For example, fiber-rich diet is associated with an increased Prevotella/Bacteroides ratios and improved glucose metabolism (100). However, diet is a highly diverse biochemical space, often tuned by the microbiota (101). Thus, dietary interventions may be personalized based on this fine-tuning of microbiome of diet (102). Zeevi et al. used an elegant strategy integrating microbiome, clinical, and dietary data to build predictive models to shape customized dietary recommendations for healthier glycaemia (18). Using genome-scale metabolic modeling, Shoaie et al. elegantly modeled the interactions of diet, gut microbiota, and host metabolism. The authors successfully predicted microbiome metabolic responses related to a dietary intervention in obese subjects and formally validated their predictions using fecal and blood metabolomics data (103). Given this microbiota plasticity, microbiome-targeted interventions are very appealing approach to personalize diet intervention. However, this microbiota plasticity may be dependent on previous dietary habits (104) and baseline microbial populations, which could hamper microbiota-directed intervention responses (105). Thus, high resolution definition of key diet-responsive microbiome is very important to enhance the prediction performance of dietary interventions. Despite current challenges that hamper effective clinical translation of microbiota-related to nutrition interventions, new analytical, and computational developments may help overcome these limits. Indeed, its integration with other omics such as proteomics and metabolomics for better and more accurate functional profiling (18, 106). However, still large-scale studies are needed to draw robust conclusions. Moreover, controlled studies are mandatory to characterize environmental factors independent of diet which may be crucial in modeling the gut microbiota ecosystem. These evidence-based requirements will help to open promising opportunities in designing personalized nutritional strategies to shape the precision nutrition era.

## Precision Nutrition Through Integrative Omics

As above mentioned, food patterns and eating behaviors are extremely complex that include multilayer informational flows (107). Gene–diet interactions might play a role in the development of and protection against chronic diseases (108). For example, the antioxidant content of the Mediterranean diet could a protective mechanism since antioxidants modulate gene expression (109). It is well known that oxidation and inflammation processes are interconnected and may contribute to different diseases the physiopathology (110). Epigenetics explores heritable DNA modifications that may regulate chromosome architecture to modulate gene expression without changing the underlying sequence (111). Epigenetic phenomena are critical for along the whole lifespan. Different studies confirm the complex interactions that exist among food components, dietary patterns, and epigenetic modifications (112, 113). Lillycrop et al. reported the effect of nutrition through the life span and how in early life nutritional exposure can induce long-term changes in DNA methylation (114). Petersen et al. reported strong influences of genetic variants on metabotypes which shows the relevant exploration of the interconnection between metabolomics and gene regulation through epigenetics (115). This confirms that integrating metabolomics to other omics might be of a great interest in both translational and clinical level. Given this biological complexity, systematically tracking relationships between diet and diseases through multiomics lences is very challenging (116, 117). So, innovative systematic strategies are urgently needed to unveil the role of food types in disease pathogenesis for, ultimately, design dietary recommendation to promote health and wellbeing or to cure diseases. Some efforts are already promising. For example, Zheng et al. proposed a computational framework for establishing diet-disease associations and additional information on the role of diet in disease development. The authors used large-scale gene expression datasets and network-based analyses to identify diet-disease relationships that could be used to build dietary recommendations tools (118). Jensen et al. proposed a platform to explore dietary recommendations in association with drugs by building food-drug association database (119). Zeevi et al. proposed an appealing and actionable integration of microbiome, clinical, and dietary data to customize dietary recommendations to tightly monitor glycemia (18). However, large-scale multi-omic nutritional intervention studies are rare. The current high cost for multi-omics large-scale dataset generation and the lack of streamlined pipelines for smooth integration of the heterogeneous datasets hamper the development of such studies.

## Challenges and Future Perspectives Toward Precision Nutrition

One of the raised limits of PN is that most studies are observational rather than from randomized controlled trials (RCTs) with assessed clinical end points. Limited information is so far available to confirm that PN produce more accurate and sustained changes in behavior than currently used approaches and do these PN strategies led to better health (7, 19, 120). So far, no large scale and long term personalized nutrition study has been carried out (19, 121). The logistical burden and costs of large-scale nutrition intervention studies with disease risk as outcomes hamper their implementation. Thus, the use of diet changes, or established biomarkers of disease (glycated hemoglobin A1c, blood pressure, or cognitive assessment) as outcomes may be potential surrogates (121). Advancement of the young discipline of PN, including metabotyping, will require new perspectives. Setting strong theoretical foundations by identifying key individual attributes that drive the personalization process. Evidence for cost effectiveness of well-designed interventional studies is also fundamental. Moreover, the introduction of a regulatory framework is mandatory to gain trust of health professionals and policy makers and enhance public protection. This will need a substantial enhancement in the scientific evidence using robust random control trials. These efforts should include multidisciplinary teams, comprising clinicians, behavioral psychologists, nutritionists, computer scientists, and biomedical scientists (19). Furthermore, the integration of other “omics” to provide deeper mechanistic insights will be crucial to improve the evidence of precision nutrition (103, 122).

## Conclusion

Metabolomics proved to be a valuable tool for the measurement of biochemical changes associated health changes related to diet. It is also, highly, promising in identification of nutritional biomarkers to monitor nutritional intervention studies. The greatest challenge for metabolomics research is its integration with other omics and phenotypic data (123). However, better standardization of metabolomics data is mandatory to effectively implement its functional and operational integration. This will enhance our knowledge of diet-health relationships. Nutrition research is intrinsically multidisciplinary, and so is metabolomics. It requires collaboration among research scientists with overlapping expertise areas including nutritionists, clinicians, nurses, bioinformaticians, analytical scientists, statisticians and chemists, and many other stakeholders. This expertise integration is vital to develop the knowledge to establish the evidence-based precision nutrition. The greatest challenge to cracking the relationships between food and health is to decipher the high inter-individual variability responses to food intake. The new frontier of the nutritional sciences lies in our ability to predictably engineer our physiologic networks for diet, health, and disease. This will ultimately allow fine tuning of diet intervention and health monitoring (18). Efforts must be done to support evidence-based nutritional research and achieve effective diet-based disease prevention.

## Author Contributions

AT performed literature search and drafted the manuscript. SB critically revised and edited the manuscript. All authors approved the final manuscript.

### Conflict of Interest Statement

The authors declare that the research was conducted in the absence of any commercial or financial relationships that could be construed as a potential conflict of interest.
